# A putative serine protease, SpSsp1, from *Saprolegnia parasitica* is recognised by sera of rainbow trout, *Oncorhynchus mykiss*

**DOI:** 10.1016/j.funbio.2014.04.008

**Published:** 2014-07

**Authors:** Kirsty L. Minor, Victoria L. Anderson, Katie S. Davis, Albert H. Van Den Berg, James S. Christie, Lars Löbach, Ali Reza Faruk, Stephan Wawra, Chris J. Secombes, Pieter Van West

**Affiliations:** a Aberdeen Oomycete Laboratory, College of Life Sciences and Medicine, Institute of Medical Sciences, University of Aberdeen, Foresterhill, Aberdeen AB25 2ZD, UK; b Scottish Fish Immunology Research Centre, Institute of Biological and Environmental Sciences, University of Aberdeen, Aberdeen AB24 2TZ, Scotland, UK; c Department of Aquaculture, Bangladesh Agricultural University, Mymensingh 2202, Bangladesh

**Keywords:** *Oncorhynchus mykiss*, Pathogenicity factor, *Saprolegnia parasitica*, Serine protease

## Abstract

Saprolegniosis, the disease caused by *Saprolegnia* sp., results in considerable economic losses in aquaculture. Current control methods are inadequate, as they are either largely ineffective or present environmental and fish health concerns. Vaccination of fish presents an attractive alternative to these control methods. Therefore we set out to identify suitable antigens that could help generate a fish vaccine against *Saprolegnia parasitica*. Unexpectedly, antibodies against *S. parasitica* were found in serum from healthy rainbow trout, *Oncorhynchus mykiss.* The antibodies detected a single band in secreted proteins that were run on a one-dimensional SDS-polyacrylamide gel, which corresponded to two protein spots on a two-dimensional gel. The proteins were analysed by liquid chromatography tandem mass spectrometry. Mascot and bioinformatic analysis resulted in the identification of a single secreted protein, SpSsp1, of 481 amino acid residues, containing a subtilisin domain. Expression analysis demonstrated that SpSsp1 is highly expressed in all tested mycelial stages of *S. parasitica*. Investigation of other non-infected trout from several fish farms in the United Kingdom showed similar activity in their sera towards SpSsp1. Several fish that had no visible saprolegniosis showed an antibody response towards SpSsp1 suggesting that SpSsp1 might be a useful candidate for future vaccination trial experiments.

## Highlights

• Sera of healthy rainbow trout have antibodies against *Saprolegnia parasitica*.
• The sera interact with a single protein from culture filtrate of *S. parasitica.*
• The antigenic protein is a secreted subtilisin-like serine protease.

## Introduction

Some of the most devastating fish infections in aquaculture are caused by oomycetes, including *Saprolegnia* and *Aphanomyces* species. *Saprolegnia parasitica* is endemic to all fresh water habitats around the world and is believed to be responsible, in part, for the decline of natural populations of salmonids globally (van West, 2006, Bruno et al., 2010). Saprolegniosis, the disease caused by *Saprolegnia* species, is characterised by grey or white fluffy patches of mycelia visible on the surface of the fish, particularly around the head, tail, and fins (Hatai & Hoshiai 1992). Infection is primarily in epidermal tissue (Fregeneda Grandes et al., 2001, Hussein and Hatai, 2002) and can, in extreme cases, cover 50 % of the fish's body. Tissue containing lesions may appear supple and ulcerated, potentially with necrotic regions, while the surrounding areas can demonstrate fluid retention and cell death (Gieseker *et al.* 2006). It has been speculated that fish infected by *S. parasitica* die from haemodilution (Richards & Pickering 1979).

Aquaculture is one of the world's fastest-growing food sectors, currently accounting for more than 50 % of total fish production (FAO 2012), with a large proportion of this coming from fresh water aquaculture (van West, 2006, FAO, 2012). Within the aquaculture industry, oomycete infections cause substantial economic losses. *Saprolegnia* species are responsible for these infections, affecting approximately one in ten hatched salmon raised in fish farms (van West 2006).

For many years, saprolegniosis was kept under control through the use of the organic dye malachite green. However, following a ban on the use of malachite green in 2002 due to potential carcinogenic effects (Srivastava et al., 2004, Sudova et al., 2007), saprolegniosis is once more prominent in aquaculture. Although the addition of salt (NaCl) to tank water has been reported to be effective in controlling saprolegniosis (Marking et al., 1994, Ali, 2005), it does not always prevent growth of *Saprolegnia* sp. nor is it considered a viable alternative to malachite green due to the large quantities that would be required in aquaculture (Marking *et al.* 1994). At present, two treatments, bronopol (Pyceze^®^, Novartis) and formalin, are often used to control saprolegniosis, however the use of formalin is currently under review due to environmental, health, and work safety considerations (EU Biocide Product Directive 2009). Therefore, it is clear that alternatives must be sought for the control of *S. parasitica* in aquaculture.

One potential route to control the disease is to develop a fish vaccine against *S. parasitica*. Vaccines are already in use in aquaculture for a range of other pathogens. For example, vaccines against bacterial diseases such as vibriosis, caused by *Vibrio anguillarum*, furunculosis, caused by *Aeromonas salmonicida* and *Vibrio ordalii*, and yersiniosis, caused by *Yersinia ruckeri*, have been routinely used for a number of years (reviewed in Gudding *et al.* 1999).

In an initial Ami-momi (Hatai and Hoshiai, 1993, Stueland et al., 2005) infection experiment of *S. parasitica* on rainbow trout, it was discovered that several fish did not become infected. In light of this observation, we decided to investigate whether secreted proteins from *S. parasitica* could be recognised by preimmune sera of both challenged and nonchallenged fish. Here we describe the response of rainbow trout sera to secreted protein fractions from *S. parasitica* and report the identification of a secreted subtilisin-like serine protease, SpSsp1.

## Materials and methods

### *Saprolegnia parasitica* culture conditions

*Saprolegnia parasitica* isolate CBS223.65, isolated from pike (*Esox lucius*), was obtained from the Centraal Bureau voor Schimmelcultures (CBS), The Netherlands. The isolate was grown routinely on Potato Dextrose Agar (Fluka) for 5 d at 18 °C, before inoculation in pea broth (125 g L^−1^ frozen peas, autoclaved, filtered through cheese cloth, volume adjusted to 1 L, pH 6.25, and autoclaved again) and incubation for 2 d at 18 or 24 °C. To accomplish *S. parasitica* sporulation, the mycelium was washed three times in sterile tap water and placed in a sterile 50:50 solution of demineralised water and aquarium tank water, obtained from a fresh water aquarium. After overnight incubation, zoospores and cysts were collected by pouring the culture filtrate through a 40 μm cell strainer and concentrated by centrifugation (5 min: 1500*g*). Germinating cysts were obtained by vortexing the zoospore/cyst suspension and incubation at 24 °C for 4–5 h. Cysts were concentrated by centrifugation (5 min: 3000*g*).

### Infection of rainbow trout with *Saprolegnia parasitica*

Ten rainbow trout (*Oncorhynchus mykiss*) (*ca* 300 g) per tank were maintained in 0.5 m^3^ flow-through, fresh water tanks (actual water volume 470 L) with a flow rate of approximately 5 L min^−1^ at a temperature of 12 °C (±2 °C). The water quality was maintained with ammonia levels of less than 0.5 mg L^−1^ and nitrite levels of less than 20 mg L^−1^. All fish were fed *ad libitum* with commercial fish pellets (Ewos).

The water level was adjusted to 150 L 1 week prior to the challenge to allow acclimatisation. Feeding of the fish was stopped 2 d before the challenge and the water supply of each tank was isolated prior to the start of the challenge. Fish were put into a net (mesh size 5 mm) and shaken in air for 2 min according to the Ami-momi technique (Hatai and Hoshiai, 1993, Stueland et al., 2005). The net containing the fish was dipped in a bucket of tank water to rinse off any mucus and the fish were released back into the challenge tank. A zoospore suspension of 3 × 10^5^ zoospores L^−1^ was carefully (to minimise encystment) added to each tank. The unchallenged negative control group underwent the Ami-momi treatment, but had no zoospore suspension added. Two days postchallenge the water flow was resumed to all tanks. Signs of infection were looked for over the next 14 d.

### Sera collection

Fish maintained as described above were anaesthetised (Benzocaine, 10 mg L^−1^) and bled from the caudal vein to obtain serum for analysis. Blood was also collected from healthy rainbow trout of approximately 200–400 g from three fish farms in Scotland immediately after they were killed. Blood was also collected from a fish with a large week-old injury, which was not showing any signs of *Saprolegnia parasitica* infection. Blood was also collected from healthy Atlantic salmon smolts (*Salmo salar*) from a further fish farm in Scotland. Blood samples were left to clot at ambient temperature then centrifuged (20 min: 3000*g*) to pellet the red blood cells. The serum was collected, aliquoted, and stored at −20 °C.

### Extraction of proteins

*Saprolegnia parasitica* strain CBS223.65 was grown for 2 d in pea broth as described above. Culture supernatant was harvested, passed through a 70 μm cell strainer to remove any mycelia fragments, collected into a 50 ml Greiner tube, and centrifuged (5 min: 1000*g*). The supernatant was precipitated in 60 % (v/v) acetone at −20 °C overnight. Subsequently, the secreted proteins were harvested by centrifugation (10 min: 13 000*g*). A 0.3 ml aliquot of 2D lysis buffer (7.5 M urea, 2.5 M thiourea, 1.25 mM EDTA (pH 8.0), 625 mM DTT, 250 mM Tris–HCl, 20 % w/v Chaps, 50 % v/v glycerol, 1× protease inhibitor (Roche), and 10 % v/v carrier ampholytes (Bio-Lyte pH 4–6)) was used to resuspend the sample pellet.

### One- and two-dimensional Sodium dodecyl sulfate-polyacrylamide gel electrophoresis (SDS-PAGE)

**Fig 1:**
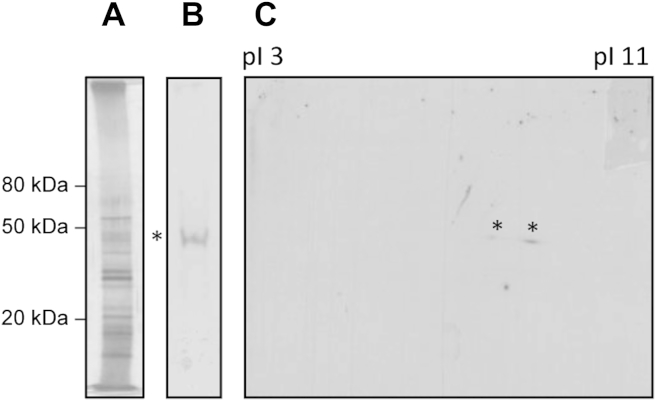
(**A**) Silver-stained gel of 1D SDS-PAGE of *Saprolegnia parasitica* secreted proteins from culture filtrate of strain C65. (**B**) Western blot of 1D SDS-PAGE of *S. parasitica* secreted proteins from culture filtrate of strain C65 (probed with sera from rainbow trout). X-ray film was exposed to the blot for 2 min. One band is recognised, indicated by an asterisk (*), corresponding to a protein of around 45 kDa. (**C**) Western blot of 2D SDS-PAGE of *S. parasitica* secreted proteins probed with sera from rainbow trout. X-ray film was exposed to the blot for 15 min (right). Two spots are recognised, indicated by two asterisks (*), corresponding to proteins of around 45 kDa.

Protein samples were denatured (3–5 min: 100 °C), centrifuged (15 s: 16 200*g*), and *ca* 50 μg protein was separated in the first dimension by isoelectric focussing (8000 V h^−1^) of 7 cm Immobiline dry polyacrylamide gel strips with an immobilized pH 3–11 nonlinear (NL) gradient using an IPGphor (Amersham Biosciences). Proteins were separated in the second dimension on Novex NuPAGE 4–12 % Bis-Tris mini-gels (Invitrogen). Gels (Fig 1C) were either stained using GelCode Blue Stain Reagent (Pierce) or silver-stained according to the method described by Kamoun *et al.* (1998).

### Immunoblotting

1D and 2D gels run as described above were transferred to nitrocellulose membranes. Each membrane was incubated at 4 °C overnight in PBS + 0.2 % Tween-20 (PBS-T) and then for 1 h in PBS-T + 10 % skimmed milk powder (MPBS-T). After washing the membrane several times with PBS-T, it was incubated for 2 h with rainbow trout sera diluted 1:100 in PBS-T at room temperature (RT). Each membrane was washed several times, followed by incubation with Horse radish peroxidase (HRP)-conjugated antitrout/antisalmon IgM antibody (Aquatic Diagnostics Ltd, Stirling) diluted 1:54 in MPBS-T. After several washes, membranes were developed by Pierce ECL Western Blotting Substrate (Thermo Scientific). Membranes were exposed to Kodak BioMax XAR film (GE Healthcare).

### ELISA

Trout serum was collected from ten individual trout belonging to one of the three different groups. Fish were immunised using (1) the recombinant SpSsp1 in adjuvant, (2) adjuvant only, and (3) saline solution. All fish were injected with a 100 μl volume. At 11 weeks postvaccination, all fish were challenged using *Saprolegnia parasitica* zoospores (see section on Infection above). Two weeks postchallenge ten fish per group were bled and serum collected and stored at −20 °C until use.

A direct ELISA protocol was adapted and applied to investigate specific antibody titres. ELISA plates (96 wells from Nunc) were coated with recombinant SpSsp1 (5–8 μg ml^−1^ in coating buffer (0.05 M carbonate/bicarbonate buffer [pH 9.6]), 50 μl per well) and left to incubate overnight at 4 °C. Unbound antigen was removed and 200 μl of ELISA blocking buffer (5 % [w/v] skimmed milk in PBS-T (PBS + 1/2000 Tween-20)) was added and incubated at RT for 2 h. The blocking buffer was subsequently removed and the plate was washed with PBS-T three times. Fifty microlitre of serum was added and dilution series (1/4 – 1/512) were made. Every sample was applied in duplicate. The plates were left to hybridize for 3 h at RT and then washed three times with PBS-T. A secondary antibody (mouse antitrout Ig (F11, Aquatic Diagnostics, Stirling, UK)) was next applied, 100 μl diluted 1/1000, and left to incubate at 37 °C for 3 h. After another wash with PBS-T the tertiary antibody goat antimouse Ig alkaline phosphatase antibody was added, 100 μl diluted 1/3000, and incubated at 37 °C for 2 h. Following a final wash step, 50 μl of alkaline phosphatase substrate solution was added and the substrate was left to develop in the dark at RT for 1 h. The plates were read at 405 nm in a spectrophotometer and readings were recorded.

### MS/MS analysis

Protein spots identified by immunoblotting were excised from the gel and digested with trypsin (sequencing grade, Promega) using an Investigator ProGest robotic workstation (Genomic Solutions Ltd.). Proteins were reduced with 10 mM Dithiothreitol (DTT) (60 °C, 20 min), S-alkylated with 50 mM iodoacetamide (25 °C, 10 min) then digested with trypsin (37 °C, 8 h). The resulting tryptic peptide extract was dried by rotary evaporation (SC110 SpeedVac; Savant Instruments) and dissolved in 0.1 % formic acid for Liquid Chromatography–Tandem Mass Spectrometry (LC–MS/MS) analysis. Peptide solutions were analysed using an HCTultra PTM Discovery System 3D ion trap (Bruker Daltonics Ltd.) coupled to an UltiMate 3000 LC System (Dionex (UK) Ltd.). Peptides were separated on a Monolithic Capillary Column (200 μm i.d. × 5 cm; Dionex) at a flow rate of 2.5 μl min^−1^ using a gradient of acetonitrile (6–38 % over 12 min) in 0.04 % (aq.) formic acid. Peptide fragment mass spectra were acquired in data-dependent AutoMS(2) mode with a scan range of 300–1500 *m*/*z*, three averages, and up to three precursor ions selected from the MS scan (100–2200 *m*/*z*). Precursors were actively excluded within a 1.0 min window. Peptide peaks were detected and deconvoluted automatically using DataAnalysis software (Bruker). Mass lists in the form of Mascot Generic Files were created automatically and used as the input for Mascot MS/MS ions searches of the *Saprolegnia parasitica* predicted protein database (downloaded from the Broad Institute website at http://www.broad.mit.edu) using the Matrix Science web server (http://www.matrixscience.com). The default search parameters used were: enzyme = trypsin; max missed cleavages = 1; fixed modifications = carbamidomethyl (C); variable modifications = oxidation (M); peptide tolerance ± 1.5 Da; MS/MS tolerance ± 0.5 Da; peptide charge = 2+ and 3+; instrument = ESI-TRAP.

### Bioinformatic analyses

The gene and protein sequences corresponding to the identified protein were downloaded from the *Saprolegnia parasitica* genome database at the Broad Institute. To identify N-terminal peptides, signal peptidase cleavage sites were predicted using the SignalP 3.0 server (http://www.cbs.dtu.dk/services/SignalP; Bendtsen *et al.* 2004) using both Eukaryotic Hidden Markov and Neural Network algorithms. The N-linked glycosylation status of SPRG_14567 was predicted using NetNGlyc (http://www.cbs.dtu.dk/services/NetNGlyc/; CBS, Denmark). The ExPASy proteomics server tool, Compute pI/Mw (http://www.expasy.org/tools/pi_tool.html; Bjellqvist *et al.* 1993) was used to obtain the theoretical pI, molecular weight, and amino acid composition of each protein, which was checked against the pI and molecular weight obtained from MS/MS. BlastP analyses were performed at the National Center for Biotechnology Information (NCBI) website (http://blast.ncbi.nlm.nih.gov/Blast.cgi; Altschul *et al.* 1997) using the nonredundant (nr) database and searching for specific domains was performed by InterProScan (http://www.ebi.ac.uk/Tools/InterProScan/). Conserved domain searches were carried out using the NCBI conserved domain database (CDD) (http://www.ncbi.nlm.nih.gov/structure/cdd/wrpsb.cgi; Marchler-Bauer *et al.* 2007). Alignment of SPRG_14567 with the subtilisin domains of the top 15 BlastP hits was performed using ClustalW2 (http://www.ebi.ac.uk/Tools/clustalw2/index.html; Larkin *et al.* 2007) and this was used as the basis for phylogenetic analysis using MEGA4 (Tamura *et al.* 2007).

### Cloning, overexpression, and enrichment of recombinant *SpSsp1*

*SpSsp1* was overexpressed and purified essentially as described by Jiang *et al.* (2013). Basically, SpSsp1^15–481^-(His)_6_ overexpressing cells were grown in modified Luria–Bertani media (tryptone 1 % w/v, yeast extract 0.5 % w/v, 100 mM NaHPO_4_, pH 7.4; 2 mM MgSO_4_, glucose 0.05 % w/v; and NaCl 0.5 % w/v) to an OD_600 nm_ of 0.6–0.8 and induced with 1 mM isopropyl-beta-D-thiogalactopyranoside (IPTG) for 3 h at 37 °C, 200 rpm. Extraction and enrichment of the SpSsp1 protein from the cells was essentially carried out as described by Jiang *et al.* (2013). Protein was checked by 1D gel electrophoresis as described above.

### SpSsp1 expression analysis

Preinfection life stages of *Saprolegnia parasitica* strain CBS223.65 were collected as described above. RNA was isolated and cDNA synthesized as described by van West *et al.* (2010) using oligo(dT) primers. Transcript levels of *SpSsp1* were analysed with a LightCycler^®^ 480 (Roche), using the LightCycler^®^ 480 Sybr green master (Roche) with 5 μl of cDNA in a total of 10 μl and according to the manufacturer's protocol. The reaction was performed with an initial incubation at 95 °C for 5 min, followed by 40 cycles of 95 °C for 10 s, 58 °C for 10 s, and 72 °C for 5 s, respectively. A dissociation curve, as described in the LightCycler^®^ 480 Sybr green master (Roche) protocol, was performed to check specificity of the primers. Disassociation curves were generated by a subsequent cycle of 95 °C for 5 s and 65 °C for 1 min. A final cooling cycle was performed at 40 °C for 10 s. The amplicon length and optimised concentrations of the primers were 140 bp and 300 nM for *SpSsp1*, respectively, and 129 bp and 400 nM for *SpTub-b*, respectively. To correct for differences in template concentration, the *SpTub-b* gene encoding for β-tubulin was used as a reference gene as suggested by van West *et al.* (2010). The primers used in the quantitative real time PCR (RT-qPCR) were as follows: for *SpSsp1* 5′-CCACGAACGAATACGTCAAG-3′ (forward) and 5′-GGTGTAGGCGTACTTGGTGA-3′ (reverse); for *SpTub-b* 5′-AGGAGATGTTCAAGCGCGTC-3′ (forward) and 5′-GATCGTTCATGTTGGACTCGGC-3′ (reverse). Subsequent analysis was performed with LightCycler^®^ 480 software (Roche), using the second derivative maximum method, which calculates and includes PCR efficiency according to Pfaffl (2001). Average values from two technical replicates of four independent RNA isolations (biological replicates) were normalised using the values from the constitutively expressed control gene (β-tubulin).

## Results

### Recognition of *Saprolegnia parasitica* secreted proteins by trout sera

Following an Ami-momi infection experiment with zoospores from *S. parasitica* (strain CBS223.65), ten trout were monitored for the development of mycelial growth consistent with saprolegniosis. Remarkably, none of the trout displayed symptoms of a successful infection. Blood was harvested from the trout 14 d after the addition of the zoospores in order to investigate whether the sera contained antibodies against potential protein antigens of *S. parasitica* following the challenge. Secreted proteins from *S. parasitica* strain CBS223.65 (three independent biological replicates) were harvested from culture filtrates of *in vitro* grown mycelium and separated by PAGE (Fig 1A). Following Western blot analysis with the pooled trout sera, a band of around 45 kDa was recognised (Fig 1B). Subsequent 2D electrophoresis and Western blotting with the trout sera resulted in the labelling of two protein spots of about 45 kDa (Fig 1C).

### Identification and bioinformatic analysis of SpSsp1

The two protein spots were excised from the Coomassie stained gels, digested with trypsin and the solubilised peptides were analysed by LC–MS/MS. The obtained MS/MS data were compared with an *in silico* digest of the *Saprolegnia parasitica* proteome, using Mascot, with a high confidence limit setting (*P* < 0.05). Both proteins spots were identified as SPRG_14567 from the genome database of *S. parasitica* on the basis of ion score and sequence coverage as predicted by Mascot MS/MS ions searches of the *S. parasitica* predicted protein database. The SPRG_14567 open reading frame (ORF) codes for a putative protein of 481 amino acids, which is in accordance with the position of the spots on the 2D gels. BlastP analyses against the NCBI database suggested that SPRG_14567 has significant sequence similarity to serine proteases from a range of oomycetes (including *Phytophthora infestans*, *Aphanomyces astaci*, *Lagenidium giganteum*, and *Pythium carolinianum*) and even bacteria (including *Beutenbergia cavernae*, *Micromonospora aurantiaca*, *Salinispora* sp., *Streptosporangium roseum*, and *Streptomyces* sp.). Furthermore, SPRG_14567 is part of a large gene family of serine proteases with 29 homologous sequences found in the genome database of *S. parasitica* of which 24, including SPRG_14567, contain a subtilase domain. Interestingly, SPRG_14567 was already described by Jiang *et al.* (2013) and found to be able to degrade trout immunoglobulin-M (IgM), demonstrating that it is an active protease.

**Fig 2:**
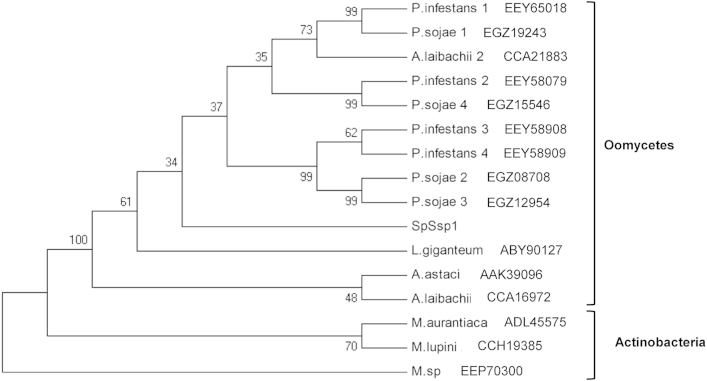
Phylogenetic relationship between the peptidase_S8_S53 domain of SpSsp1 and selected serine proteases. Serine proteases obtained by BlastP analysis of SpSsp1 against the nr protein database in NCBI. The phylogenetic tree was constructed using the maximum parsimony methods based on the peptidase_S8_S53 domain of the serine protease sequences. Bootstrap values on the consensus tree were inferred from 1000 replicates, with percentile values indicated at the nodes. SpSsp1: *Saprolegnia parasitica* CBS233.65, aa 155–404 (SPRG_14567); A.astaci_AAK39096: *Aphanomyces astaci* subtilisin-like serine proteinase precursor, aa 179–421 (Accession no: AAK39096); A.laibachii_1_CCA16972: *Albugo laibachii* Nc14 serine protease family S08A, putative, aa 475–730 (CCA16972); A.laibachii_2_CCA21883: *A. laibachii* Nc14 serine protease family S08A, putative, aa 162–399 (CCA21883); P.infestans_1_EEY65018: *Phytophthora infestans* serine protease family S08A, putative, aa 187–425 (EEY65018); P.infestans_2_EEY58079: *P. infestans* serine protease family S08A, putative, aa 141–385 (EEY58079); P.infestans_3_EEY58908: *P. infestans* serine protease family S08A, putative, aa 195–433 (EEY58908); P.infestans_4_EEY58909: *P. infestans* serine protease family S08A, putative, aa 196–399 (EEY58909); P.sojae_1_EGZ19243: *Phytophthora sojae* subtilisin serine protease, aa 191–428 (EGZ19243); P.sojae_2_EGZ08708: *P. sojae* subtilisin serine protease, aa 132–382 (EGZ08708); P.sojae_3_EGZ12954: *P. sojae* subtilisin serine protease, aa 184–434 (EGZ12954); P.sojae_4_EGZ15546: *P. sojae* hypothetical protein PHYSODRAFT_509390, aa 143–396 (EGZ15546); L.giganteum_ABY90127: *Lagenidium giganteum* subtilisin-like serine protease, aa 1–174 (ABY90127); M.sp_ZY_04604370: *Micromonospora* sp. ATCC 39149 peptidase S8 and S53 subtilisin kexin sedolisin, aa 191–444 (EEP70300); M.lupini_ZP_21031234: *Micromonospora lupini* str. Lupac 08Peptidase S8 and S53 subtilisin kexin sedolisin, aa 194–454 (CCH19385); M.aurantiaca_YP_003835151: *Micromonospora aurantiaca* peptidase S8 and S53 subtilisin kexin sedolisin, aa 191–451 (ADL45575).

The presence of a signal peptide in SPRG_14567 (amino acids 1–17) was also predicted (SignalP), suggesting that the protein is indeed secreted as expected, since it was detected in culture filtrate of *S. parasitica* growing mycelium. Therefore the protein was named SpSsp1 (*S. parasitica* secreted serine protease 1). NetNGlyc reported positive results for the amino acid sequence of SpSsp1, with a predicted N-glycosylation site on reside 231 (potential: 0.69; jury agreement: 9/9). A second N-linked glycosylation site is predicted at residue 434, however the potential and jury agreement for this site are low and may represent a false positive (0.51 and 5/9, respectively). Analysis of the predicted protein sequence for the presence of conserved domains revealed the presence of the peptidases_S8_S53 superfamily domain (*E* value 6.38e^−80^) with the conserved Asp/His/Ser catalytic triad (indicated in Fig S1). Analysis by InterProScan confirmed the presence of the superfamily domain predicted by the NCBI CDD. The peptidase domain of SpSsp1 was aligned to those of the top 15 BlastP hits from the NCBI nr database (Fig S2) and can be seen to cluster with other oomycete sequences in the phylogenetic tree (Fig 2).

### Transcription analysis of SpSsp1 throughout the life stages of *Saprolegnia parasitica*

**Fig 3:**
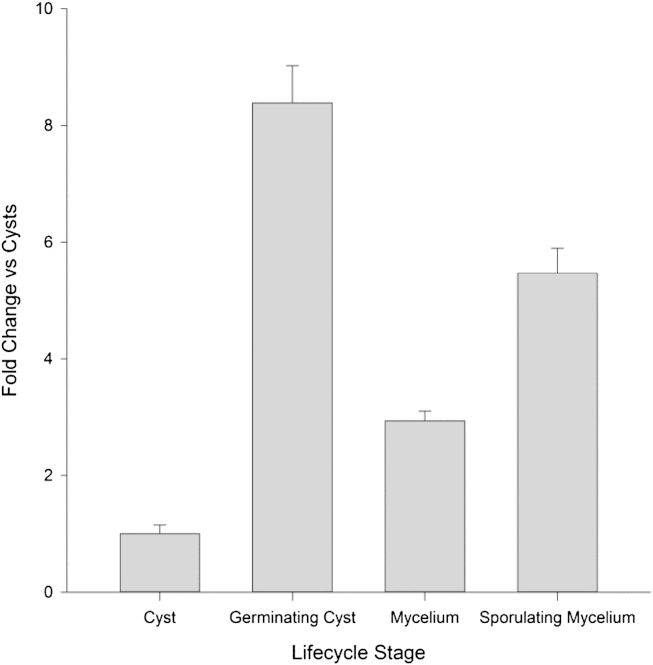
Transcript levels of *SpSsp1* throughout the life stages of *Saprolegnia parasitica*. Transcript levels are relative to those of *SpSsp1* in cysts and normalised against the reference gene *SpTub-b* encoding tubulin. Error bars correspond to standard error of four biological replicates.

RT-qPCR was carried out on cDNA samples from various life cycle stages of *S. parasitica* to obtain a detailed expression profile of *SpSsp1* (Fig 3). The constitutively expressed tubulin gene, *SpTub-b*, was used as a reference gene and the expression of *SpSsp1* in cysts was set to 1 to allow comparison with other life stage samples. Expression of *SpSsp1* is upregulated around five-fold in sporulating mycelia compared to cysts rising further to around eight-fold upregulation in germinating cysts. Vegetative mycelia also show increased mRNA levels, that are about three-fold higher than in cysts.

### Recognition of recombinant SpSsp1 protein by the trout sera

**Fig 4:**
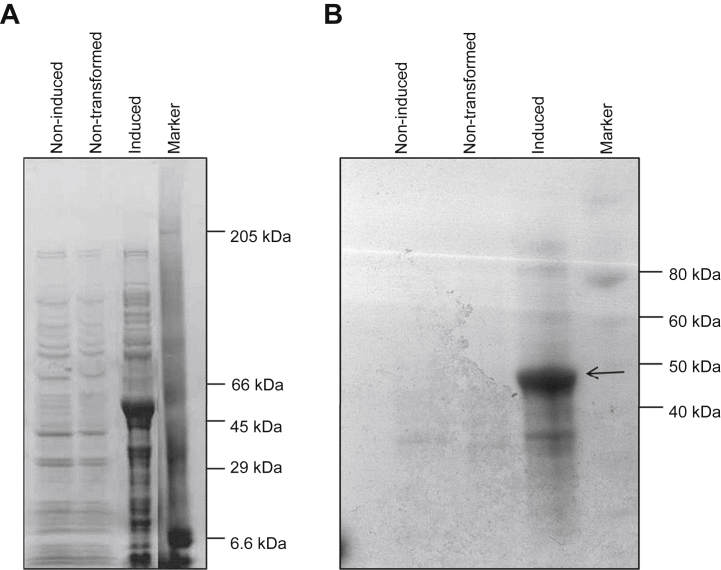
Serum from trout recognises recombinant SpSsp1^15–481^. (**A**) Coomassie stained 1D SDS-PAGE of proteins obtained from 1 ml of *Escherichia coli* culture resuspended in protein sample buffer. In the sample where the SpSsp1^15–481^(His)_6_ expression was induced one band around 40–45 kDa was specifically detected (lane 3). (**B**) Western blot of 1D SDS-PAGE of proteins obtained from 1 ml of *E. coli* culture resuspended in protein sample buffer, probed with sera from fish maintained in the fresh water aquarium facilities at the School of Biological Sciences, University of Aberdeen. X-ray film was exposed to the blot for 1 min. Several proteins from *E. coli* are recognised in all three sample lanes, however recombinant SpSsp1^18–…^(His)_6_ is specifically recognised (indicated by an arrow) at around 40–45 kDa.

In order to confirm that SpSsp1 is the protein recognised by the immune serum of the trout, as was described above (Fig 1), a recombinant and His-tagged (C-terminal) fusion construct of SpSsp1, without the first 14 amino acids of the signal peptide (SpSsp1^15–481^-His) was produced (Fig 4A) and partly purified from an *Escherichia coli* overexpressing strain, as described recently by Jiang *et al.* (2013). SpSsp1^15–481^-His was run on a 1D gel, blotted, and incubated with serum isolated from the trout (Fig 4B). The serum was able to bind to SpSsp1^15–481^-His, demonstrating that this was the *Saprolegnia parasitica* antigen recognised in the trout serum.

### *SpSsp1* is recognised in trout sera from rainbow trout cultured in fish farms

**Fig 5:**
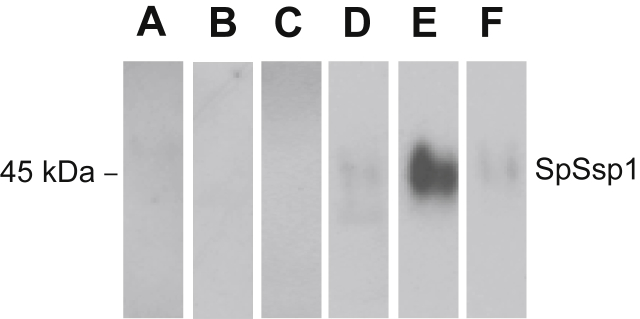
Western blot of SpSsp1 incubated with serum obtained from trout and salmon from fish farms in the UK. The same amount of enriched SpSsp1 was loaded in each lane and run on an SDS-PAGE gel, blotted and a Western blot was performed with sera of six individual fish from several fish farms. (**A**) Serum from a random salmon harvested from a farm in Scotland. (**B**–**F**) Serum from rainbow trout obtained from three independent trout farms in the UK (**B**–**E**). (**E**) A severely wounded trout, which did not show any visual symptoms of saprolegniosis. (**F**) Rainbow trout maintained in the fresh water aquarium facilities at the University of Aberdeen. The X-ray film was exposed to blots (**A**–**D**) and (**F**) for 2 min and blot (**E**) for 20 s. There is no recognition of recombinant SpSsp1 protein by sera samples from the salmon (**A**), and the trout of two fish farms (**B** & **C**), weak recognition of protein by sera from the third fish farm (**D**) and the aquarium facilities at the University of Aberdeen (**F**). Serum of the injured fish from farm (**E**) showed a strong response towards SpSsp1.

We then wanted to investigate whether trout and salmon (*Salmo salar*) kept in several fish farms across the UK (South of England, South Scotland, and the West Coast of Scotland) showed a similar immune response towards the serine protease from *Saprolegnia parasitica*. Immuno blots, utilising trout sera collected from three fish farms in Scotland, showed no or faint recognition of SpSsp1^15–481^-His, similar to the response of trout kept at the University of Aberdeen aquarium facility (Fig 5). However, one particular fish that was injured by a cormorant, about a week prior to blood sampling showed a particularly strong immune response towards SpSsp1^15–481^-His. The serum of a random salmon that was harvested for an unrelated project was also tested but was not detecting SpSsp1^15–481^-His.

### Immunisation of trout with *SpSsp1*

**Fig 6:**
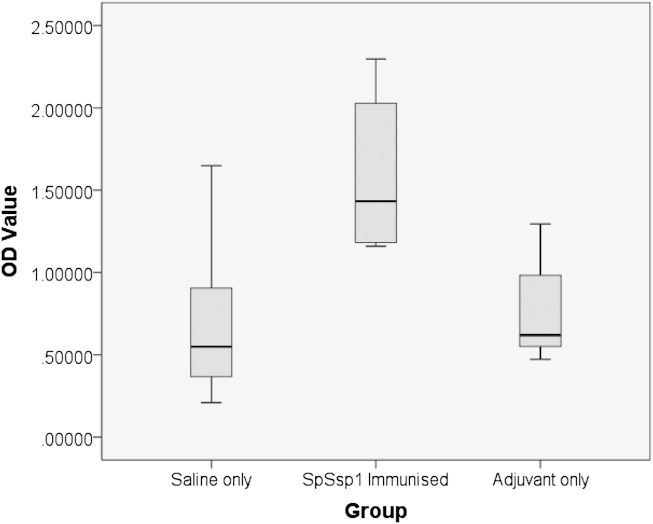
SpSsp1-immunised trout show an increase in antibody production towards SpSsp1 as determined by ELISA. Trout injected with a saline solution or adjuvant only showed similar levels of antibody production. Fish immunised with SpSsp1 in adjuvant showed a significantly higher antibody response (*P* < 0.001). Each group consists of samples taken from ten individual trout.

The formation of specific antibodies against SpSsp1 upon vaccination in groups of ten fish was investigated using an ELISA protocol (Fig 6). The ELISA experiment showed that injection of SpSsp1 in fish resulted in a higher titre of antibodies against SpSsp1 than the control groups (adjuvant alone or saline), suggesting that an immune response was initiated against the antigen.

## Discussion

The present study identifies the presence of serum antibodies against *Saprolegnia parasitica* in rainbow trout. This finding is in agreement with results obtained by Fregeneda-Grandes *et al.* (2009), who have also found specific antibodies in the serum of healthy and infected wild brown trout (*Salmo trutta* L.) and in the serum of brown trout following injection with antigenic extracts from *S. parasitica* (Fregeneda Grandes *et al.* 2007). They observed significantly higher prevalence of *S. parasitica* antibodies in trout sera from areas of high reported incidences of saprolegniosis compared with areas where incidences of saprolegniosis are relatively low (Fregeneda-Grandes *et al.* 2009). Unfortunately, they did not investigate the putative function of the two main antigenic proteins (with approximate sizes of 25 kDa and 29 kDa) in their study. Presumably SpSsp1 is a different antigen as it has a molecular size of 45 kDa. Furthermore, Hodkinson & Hunter (1970) found that 93 % of wild salmon suspected of ulcerative dermal necrosis (UDN) infection had antibodies against *Saprolegnia*, despite only 66 % demonstrating signs of saprolegniosis. A link between the presence of antibodies against *S. parasitica* and lack of saprolegniosis was also observed in our study in an injured rainbow trout (Fig 5E) that was recovering from a recent wound inflicted by a cormorant. Fregeneda-Grandes *et al.* (2009) also suggested a link between antibody presence and incidence of saprolegniosis. In their study a lower prevalence of antibodies was observed in *Saprolegnia*-infected trout (18 %) suggesting either potential immune suppression or inability to raise an appropriate specific immune response. Taken together, these observations hint that the adaptive immune system does play a role in defence against *Saprolegnia*.

Recently we found that the innate immune response in fish can also be activated during infection with *S. parasitica*, since proinflammatory cytokine transcripts were induced in four different cell lines infected with *S. parasitica* (de Bruijn *et al.* 2012). It was found that IL-1β1, IL-8, IL-11, TNF-α2, COX-2, the acute phase protein serum amyloid A, C-type lectins CD209a and CD209b were all upregulated during infection. Furthermore several antimicrobial peptides (AMPs) were also upregulated in response to *Saprolegnia* infection, including hepcidin and cathelicidin 1 (rtCATH1) and 2 (rtCATH2). Interestingly, rtCATH2 was also able to delay sporulation in *S. parasitica* (de Bruijn *et al.* 2012). Also a strong upregulation of the proinflammatory genes COX-2, IL-1β, and TNFα was observed in a monocyte/macrophage cell line of trout (RTS11) in response to an infection with *Achlya* (Kales *et al.* 2007). By employing microarray analysis of juvenile Atlantic salmon, Roberge *et al.* (2007) showed that several immune genes, including components of the complement system, C-type lectin receptor, a CD209-like protein, and TAP2, were induced upon *Saprolegnia* infection.

The ubiquitous nature of *S. parasitica* in the fresh water environment makes it very likely that fish in farms and wild fish are continuously exposed to this pathogen and that such interactions could explain the presence of the antibodies against SpSsp1 described in this study. The serum antibodies recognised two distinct spots following 2D SDS-PAGE, which were subsequently identified by bioinformatic analysis as a single protein, SpSsp1. The function of SpSsp1 is predicted to be a subtilisin-like serine protease due to significant similarity to serine proteases from other organisms as well as the presence of the canonical subtilisin aspartic acid/histidine/serine catalytic triad.

Subtilisin and other serine proteases have long been implicated as pathogenicity factors for bacteria and true fungi. Paton *et al.* (2006) found that the subtilase cytotoxin from *Escherichia coli* cleaves the essential endoplasmic reticulum chaperone, BiP, resulting in cell death. Kolattukudy *et al.* (1993) identified an elastolytic serine protease, which was subsequently identified as a significant virulence factor of *Aspergillus fumigatus*. Mutants defective in the production of this protein caused dramatically reduced mortality in the host compared to the wild type strain. A zinc metalloprotease from *Vibrio aestuarianus* has been shown to impair host immune responses in the Pacific oyster, *Crassostrea gigas* (Labreuche *et al.* 2010), while in the major cabbage pathogen *Plasmodiophora brassicae*, a serine protease (Pro1) has been identified that stimulates resting spore germination through proteolytic activity (Feng *et al.* 2010). Interestingly, Jiang *et al.* (2013) found that metalloproteinases and serine proteases secreted by *S. parasitica* are capable of degrading trout immunoglobulin IgM, and that enriched SPRG_14567 (SpSsp1) could degrade trout IgM, suggesting this protein is a potential virulence factor with a role in suppression of host immune responses. Considering this potential role of the protein in degradation of fish IgM, it is interesting that sera from apparently *Saprolegnia*-resistant trout show recognition of SpSsp1. This could possibly indicate that immunological recognition of SpSsp1 leads to a sufficient adaptive immune response in the fish that is able to resist *Saprolegnia* infection.

Indeed, after immunising trout with SpSsp1, we could demonstrate that SpSsp1 is immunogenic since an increase in antibody production against SpSsp1 was detected *via* ELISA. However, at this stage, it should be noted that we cannot rule out, the possibility that the fish initially mounted an immune response to a related molecule such as a bacterial derived serine protease, since SpSsp1 has significant similarity to serine proteases from bacteria living in sediments (*Salinispora* sp.) and fresh water (*Micromonospora* sp.). SpSsp1 also has significant similarity to a putative subtilisin-like serine protease from the fish pathogenic actinobacteria *Streptomyces griseus*. The high level of similarity between SpSsp1 and bacterial subtilisin-like proteases, particularly in the subtilisin domain, raises the possibility that the trout could also have encountered a bacterial subtilisin to which it raised antibodies that cross react with SpSsp1.

Transcript analysis of *SpSsp1* showed expression in all life stages with the greatest expression in germinating cysts compared to cysts. Whilst this data differs to the initial RNA-seq data reported by Jiang *et al.* (2013), where highest expression was in mycelium rather than germinated cysts, slight variations in culturing and harvesting conditions and/or the higher reliability of RT-qPCR analysis *versus* RNA-seq data may account for this difference. Indeed, considering the potential role of this protein in evasion of host cell defences, expression of this protein in life stages where *S. parasitica* is interacting with host cells would increase the likelihood of successful infection.

In conclusion, the present study identifies a secreted serine protease from *S. parasitica* that appears to be recognised by antibodies in trout serum. Interestingly, the trout that we analysed and that contain these antibodies could not be infected following the Ami-moni infection technique. Therefore we would like to speculate that the immunological recognition of SpSsp1 from *S. parasitica* by fish might give protection to this disease. Future studies will focus on the role of SpSsp1 in infection and the potential for its use as a vaccine against *Saprolegnia*.

## Appendix A Supplementary data

Fig S1 (**A**) Protein sequence of SPRG_14567 (SpSsp1). Signal peptide according to SignalP is underlined and the conserved Asp/His/Ser catalytic triad, identified by the NCBI Conserved Domain Database is **bold** and italic. Potential N-glycosylation sites are indicated in bold. (**B**) Genomic sequence of SPRG_14567 (SpSsp1). Coding sequence indicated in **bold**.

Fig S2 Alignment of the peptidase_S8_S53 domains of SpSsp1 with those of the top 15 serine proteases obtained by BlastP analysis of SpSsp1 against nonredundant protein database in NCBI. SpSsp1: *Saprolegnia parasitica* CBS233.65, aa 155–404 (SPRG_14567); A.astaci_AAK39096: *Aphanomyces astaci* subtilisin-like serine proteinase precursor, aa 179–421 (Accession no: AAK39096); A.laibachii_1_CCA16972: *Albugo laibachii* Nc14 serine protease family S08A, putative, aa 475–730 (CCA16972); A.laibachii_2_CCA21883: *A. laibachii* Nc14 serine protease family S08A, putative, aa 162–399 (CCA21883); P.infestans_1_XP_002897506: *Phytophthora infestans* serine protease family S08A, putative, aa 187–425 (XP002897506); P.infestans_2_XP_002909265: *P. infestans* serine protease family S08A, putative, aa 141–385 (XP_002909265); P.infestans_3_XP_002901381: *P. infestans* serine protease family S08A, putative, aa 195–433 (XP002901381); P.infestans_4_XP_002901382: *P. infestans* serine protease family S08A, putative, aa 196–399 (XP_002901382); P.sojae_1_EGZ19243: *Phytophthora sojae* subtilisin serine protease, aa 191–428 (EGZ19243); P.sojae_2_EGZ08708: *P. sojae* subtilisin serine protease, aa 132–382 (EGZ08708); P.sojae_3_EGZ12954: *P. sojae* subtilisin serine protease, aa 184–434 (EGZ12954); P.sojae_4_EGZ15546: *P. sojae* hypothetical protein PHYSODRAFT_509390, aa 143–396 (EGZ15546); L.giganteum_ABY90127: *Lagenidium giganteum* subtilisin-like serine protease, aa 1–174 (ABY90127); M.sp_ZY_04604370: *Micromonospora* sp. ATCC 39149 peptidase S8 and S53 subtilisin kexin sedolisin, aa 191–444 (ZP04604370); M.lupini_ZP_21031234: *Micromonospora lupini* str. Lupac 08Peptidase S8 and S53 subtilisin kexin sedolisin, aa 194–454 (ZP21031234); M.aurantiaca_YP_003835151: *Micromonospora aurantiaca* peptidase S8 and S53 subtilisin kexin sedolisin, aa 191–451 (YP003835151). ‘*’ indicates that the residues or nucleotides in that column are identical in all sequences in the alignment; ‘:’ indicates that conserved substitutions have been observed, according to the physicochemical properties as described for ClustalW; ‘.’ indicates that semiconserved substitutions are observed.

## References

[bib1] Ali E.H. (2005). Morphological and biochemical alterations of oomycete fish pathogen *Saprolegnia parasitica* as affected by salinity, ascorbic acid and their synergistic action. Mycopathologia.

[bib2] Altschul S.F., Madden T.L., Schäffer A.A., Zhang J., Zhang Z., Miller W., Lipman D.J. (1997). Gapped BLAST and PSI-BLAST: a new generation of protein database search programs. Nucleic Acids Research.

[bib3] Bendtsen J.D., Nielsen H., Von Heijne G., Brunak S. (2004). Improved prediction of signal peptides: SignalP 3.0. Journal of Molecular Biology.

[bib4] Bjellqvist B., Hughes G.J., Pasquali C., Paquet N., Ravier F., Sanchez J.C., Frutiger S., Hochstrasser D. (1993). The focusing positions of polypeptides in immobilized pH gradients can be predicted from their amino acid sequences. Electrophoresis.

[bib5] Bruno D.W., van West P., Beakes G.W., Woo P.T.K., Bruno D.W. (2010).

[bib6] de Bruijn I., Belmonte R., Anderson V.L., Saraiva M., Wang T., van West P., Secombes C.J. (2012). Immune gene expression in trout cell lines infected with the fish pathogenic oomycete *Saprolegnia parasitica*. Developmental and Comparative Immunology.

[bib7] EU Biocide Product Directive (2009). http://ec.europa.eu/environment/biocides/index.htm.

[bib8] FAO (2012).

[bib9] Feng J., Hwang R., Hwang S.F., Strelkov S.E., Gossen B.D., Zhou Q.X., Peng G. (2010). Molecular characterization of a serine protease Pro1 from *Plasmodiophora brassicae* that stimulates resting spore germination. Molecular Plant Pathology.

[bib10] Fregeneda Grandes J.M., Fernández Díez M., Aller Gancedo J.M. (2001). Experimental pathogenicity in rainbow trout, *Oncorhynchus mykiss* (Walbaum), of two distinct morphotypes of long-spined *Saprolegnia* isolates obtained from wild brown trout, *Salmo trutta* L., and river water. Journal of Fish Diseases.

[bib11] Fregeneda Grandes J.M., RodrÍ́guez-Cadenas F., Carbajal-González M.T., Aller-Gancedo J.M. (2007). Antibody response of brown trout *Salmo trutta* injected with pathogenic *Saprolegnia parasitica* antigenic extracts. Diseases of Aquatic Organisms.

[bib12] Fregeneda-Grandes J.M., Carbajal-González M.T., Aller-Gancedo J.M. (2009). Prevalence of serum antibodies against *Saprolegnia parasitica* in wild and farmed brown trout *Salmo trutta*. Diseases of Aquatic Organisms.

[bib13] Gieseker C.M., Serfling S.G., Reimschuessel R. (2006). Formalin treatment to reduce mortality associated with *Saprolegnia parasitica* in rainbow trout, *Oncorhynchus mykiss*. Aquaculture.

[bib14] Gudding R., Lillehaug A., Evensen Ø. (1999). Recent developments in fish vaccinology. Veterinary Immunology and Immunopathology.

[bib15] Hatai K., Hoshiai G. (1992). Mass mortality in cultured coho salmon (*Oncorhynchus kisutch*) due to *Saprolegnia parasitica* coker. Journal of Wildlife Diseases.

[bib16] Hatai K., Hoshiai G. (1993). Characteristics of two *Saprolegnia* species isolated from *Coho salmon* with saprolegniosis. Journal of Aquatic Animal Health.

[bib17] Hodkinson M., Hunter A. (1970). Immune response of U.D.N.-infected salmon to *Saprolegnia*. Journal of Fish Biology.

[bib18] Hussein M.M.A., Hatai K. (2002). Pathogenicity of *Saprolegnia* species associated withoutbreaks of salmonid saprolegniosis in Japan. Fisheries Science.

[bib19] Jiang R.H.J., de Bruijn I., Haas B.J., Belmonte R., Löbach L., Christie J., van den Ackerveken G., Bottin A., Bulone V., Díaz-Moreno S.M., Dumas B., Fan L., Gaulin E., Govers F., Grenville-Briggs L.J., Horner N.R., Levin J.Z., Mammella M., Meijer H.J.G., Morris M., Nusbaum C., Oome S., Phillips A.J., Rzeszutek E., Saraiva M., Secombes C.J., Seidl M., Snel B., Stassen J.H.M., Sykes S., Tripathy S., van den Berg A.H., van Rooyen D., Vega-Arreguin J.C., Wawra S., Young S., Dieguez-Uribeondo J., Russ C., Tyler B.M., van West P., Broad Genome Annotation Group (2013). Distinctive repertoire of potential virulence genes in the genome of the oomycete fish pathogen *Saprolegnia parasitica*. PLOS-Genetics.

[bib20] Kales S.C., DeWitte-Orr S.J., Bols N.C., Dixon B. (2007). Response of the rainbow trout monocyte/macrophage cell line, RTS11 to the water molds *Achlya* and *Saprolegnia*. Molecular Immunology.

[bib21] Kamoun S., van West P., Vleeshouwers V.G.A.A., de Groot K.E., Govers F. (1998). Resistance of *Nicotiana benthamiana* to *Phytophthora infestans* is mediated by the recognition of the elicitor protein INF1. The Plant Cell.

[bib22] Kolattukudy P.E., Lee J.D., Rogers L.M., Zimmerman P., Ceselski S., Fox B., Stein B., Copelan E.A. (1993). Evidence for possible involvement of an elastolytic serine protease in aspergillosis. Infection and Immunity.

[bib23] Labreuche Y., Le Roux F., Henry J., Zatylny C., Huvet A., Lambert C., Soudant P., Mazel D., Nicolas J.-L. (2010). *Vibrio aestuarianus* zinc metalloprotease causes lethality in the Pacific oyster *Crassostrea gigas* and impairs the host cellular immune defenses. Fish & Shellfish Immunology.

[bib24] Larkin M.A., Blackshields G., Brown N.P., Chenna R., McGettigan P.A., McWilliam H., Valentin F., Wallace I.M., Wilm A., Lopez R., Thompson J.D., Gibson T.J., Higgins D.G. (2007). Clustal W and Clustal X version 2.0. Bioinformatics.

[bib25] Marchler-Bauer A., Anderson J.B., Derbyshire M.K., DeWeese-Scott C., Gonzales N.R., Gwadz M., Hao L., He S., Hurwitz D.I., Jackson J.D., Ke Z., Krylov D., Lanczycki C.J., Liebert C.A., Liu C., Lu F., Lu S., Marchler G.H., Mullokandov M., Song J.S., Thanki N., Yamashita R.A., Yin J.J., Zhang D., Bryant S.H. (2007). CDD: a conserved domain database for interactive domain family analysis. Nucleic Acids Research.

[bib26] Marking L.L., Rach J.J., Schreier T.M. (1994). American fisheries society evaluation of antifungal agents for fish culture. The Progressive Fish-Culturist.

[bib27] Pfaffl M.W. (2001). A new mathematical model for relative quantification in real-time RT-PCR. Nucleic Acids Research.

[bib28] Paton A.W., Beddoe T., Thorpe C.M., Whisstock J.C., Wilce M.C.J., Rossjohn J., Talbot U.M., Paton J.C. (2006). AB5 subtilase cytotoxin inactivates the endoplasmic reticulum chaperone BiP. Nature.

[bib29] Richards R.H., Pickering A.D. (1979). Changes in serum parameters of *Saprolegnia*-infected brown trout *Salmo trutta* L. Journal of Fish Diseases.

[bib30] Roberge C., Paez D.J., Rossignol O., Guderley H., Dodson J., Bernatchez L. (2007). Genome-wide survey of the gene expression response to saprolegniasis in Atlantic salmon. Molecular Immunology.

[bib31] Srivastava S., Sinha R., Roy D. (2004). Toxicological effects of malachite green. Aquatic Toxicology.

[bib32] Stueland S., Hatai K., Skaar I. (2005). Morphological and physiological characteristics of *Saprolegnia* spp. strains pathogenic to Atlantic salmon, *Salmo salar* L. Journal of Fish Diseases.

[bib33] Sudova E., Machova J., Svobodova Z., Vesely T. (2007). Negative effects of malachite green and possibilities of its replacement in the treatment of fish eggs and fish: a review. Veterinarni Medicina.

[bib34] Tamura K., Dudley J., Nei M., Kumar S. (2007). MEGA4: Molecular Evolutionary Genetics Analysis (MEGA) software version 4.0. Molecular Biology and Evolution.

[bib35] van West P. (2006). *Saprolegnia parasitica*, an oomycete pathogen with a fishy appetite: new challenges for an old problem. Mycologist.

[bib36] van West P., De Bruijn I., Minor K.L., Phillips A.J., Robertson E.J., Wawra S., Bain J., Anderson V.L., Secombes C.J. (2010). The putative RxLR effector protein SpHtp1 from the fish pathogenic oomycete *Saprolegnia parasitica* is translocated into fish cells. FEMS Microbiology Letters.

