# Supplementary material for: A putative serine protease, SpSsp1, from Saprolegnia parasitica is recognised by sera of rainbow trout, Oncorhynchus mykiss
Source: Fungal Biol. 2014 Jul;118(7):630–9. doi: 10.1016/j.funbio.2014.04.008 (PMC4152625; doi:10.1016/j.funbio.2014.04.008)
Supplement: Fig S1 — (A) Protein sequence of SPRG_14567 (SpSsp1). Signal peptide according to SignalP is underlined and the conserved Asp/His/Ser catalytic triad, identified by the NCBI Conserved Domain Database is bold and italic. Potential N-glycosylation sites are indicated in bold. (B) Genomic sequence of SPRG_14567 (SpSsp1). Coding sequence indicated in bold. [file mmc1.pptx]

## Slide 1
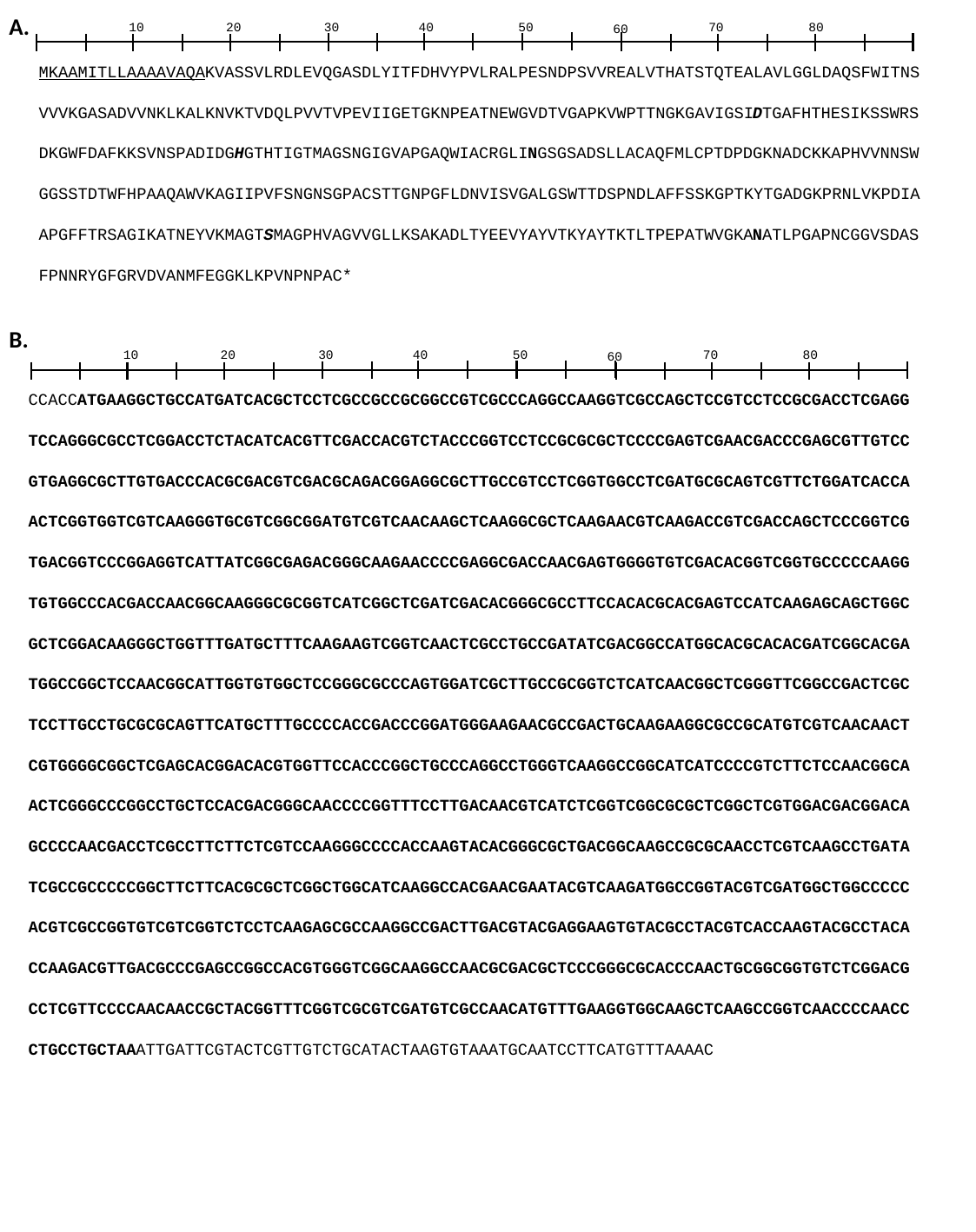

A.
40
10
20
30
50
70
80
60
MKAAMITLLAAAAVAQAKVASSVLRDLEVQGASDLYITFDHVYPVLRALPESNDPSVVREALVTHATSTQTEALAVLGGLDAQSFWITNS
VVVKGASADVVNKLKALKNVKTVDQLPVVTVPEVIIGETGKNPEATNEWGVDTVGAPKVWPTTNGKGAVIGSIDTGAFHTHESIKSSWRS
DKGWFDAFKKSVNSPADIDGHGTHTIGTMAGSNGIGVAPGAQWIACRGLINGSGSADSLLACAQFMLCPTDPDGKNADCKKAPHVVNNSW
GGSSTDTWFHPAAQAWVKAGIIPVFSNGNSGPACSTTGNPGFLDNVISVGALGSWTTDSPNDLAFFSSKGPTKYTGADGKPRNLVKPDIA
APGFFTRSAGIKATNEYVKMAGTSMAGPHVAGVVGLLKSAKADLTYEEVYAYVTKYAYTKTLTPEPATWVGKANATLPGAPNCGGVSDAS
FPNNRYGFGRVDVANMFEGGKLKPVNPNPAC*
B.
40
10
20
30
50
70
80
60
CCACCATGAAGGCTGCCATGATCACGCTCCTCGCCGCCGCGGCCGTCGCCCAGGCCAAGGTCGCCAGCTCCGTCCTCCGCGACCTCGAGG
TCCAGGGCGCCTCGGACCTCTACATCACGTTCGACCACGTCTACCCGGTCCTCCGCGCGCTCCCCGAGTCGAACGACCCGAGCGTTGTCC
GTGAGGCGCTTGTGACCCACGCGACGTCGACGCAGACGGAGGCGCTTGCCGTCCTCGGTGGCCTCGATGCGCAGTCGTTCTGGATCACCA
ACTCGGTGGTCGTCAAGGGTGCGTCGGCGGATGTCGTCAACAAGCTCAAGGCGCTCAAGAACGTCAAGACCGTCGACCAGCTCCCGGTCG
TGACGGTCCCGGAGGTCATTATCGGCGAGACGGGCAAGAACCCCGAGGCGACCAACGAGTGGGGTGTCGACACGGTCGGTGCCCCCAAGG
TGTGGCCCACGACCAACGGCAAGGGCGCGGTCATCGGCTCGATCGACACGGGCGCCTTCCACACGCACGAGTCCATCAAGAGCAGCTGGC
GCTCGGACAAGGGCTGGTTTGATGCTTTCAAGAAGTCGGTCAACTCGCCTGCCGATATCGACGGCCATGGCACGCACACGATCGGCACGA
TGGCCGGCTCCAACGGCATTGGTGTGGCTCCGGGCGCCCAGTGGATCGCTTGCCGCGGTCTCATCAACGGCTCGGGTTCGGCCGACTCGC
TCCTTGCCTGCGCGCAGTTCATGCTTTGCCCCACCGACCCGGATGGGAAGAACGCCGACTGCAAGAAGGCGCCGCATGTCGTCAACAACT
CGTGGGGCGGCTCGAGCACGGACACGTGGTTCCACCCGGCTGCCCAGGCCTGGGTCAAGGCCGGCATCATCCCCGTCTTCTCCAACGGCA
ACTCGGGCCCGGCCTGCTCCACGACGGGCAACCCCGGTTTCCTTGACAACGTCATCTCGGTCGGCGCGCTCGGCTCGTGGACGACGGACA
GCCCCAACGACCTCGCCTTCTTCTCGTCCAAGGGCCCCACCAAGTACACGGGCGCTGACGGCAAGCCGCGCAACCTCGTCAAGCCTGATA
TCGCCGCCCCCGGCTTCTTCACGCGCTCGGCTGGCATCAAGGCCACGAACGAATACGTCAAGATGGCCGGTACGTCGATGGCTGGCCCCC
ACGTCGCCGGTGTCGTCGGTCTCCTCAAGAGCGCCAAGGCCGACTTGACGTACGAGGAAGTGTACGCCTACGTCACCAAGTACGCCTACA
CCAAGACGTTGACGCCCGAGCCGGCCACGTGGGTCGGCAAGGCCAACGCGACGCTCCCGGGCGCACCCAACTGCGGCGGTGTCTCGGACG
CCTCGTTCCCCAACAACCGCTACGGTTTCGGTCGCGTCGATGTCGCCAACATGTTTGAAGGTGGCAAGCTCAAGCCGGTCAACCCCAACC
CTGCCTGCTAAATTGATTCGTACTCGTTGTCTGCATACTAAGTGTAAATGCAATCCTTCATGTTTAAAAC
