# Supplementary material for: A putative serine protease, SpSsp1, from Saprolegnia parasitica is recognised by sera of rainbow trout, Oncorhynchus mykiss
Source: Fungal Biol. 2014 Jul;118(7):630–9. doi: 10.1016/j.funbio.2014.04.008 (PMC4152625; doi:10.1016/j.funbio.2014.04.008)
Supplement: Fig S2 — Alignment of the peptidase_S8_S53 domains of SpSsp1 with those of the top 15 serine proteases obtained by BlastP analysis of SpSsp1 against nonredundant protein database in NCBI. SpSsp1: Saprolegnia parasitica CBS233.65, aa 155–404 (SPRG_14567); A.astaci_AAK39096: Aphanomyces astaci subtilisin-like serine proteinase precursor, aa 179–421 (Accession no: AAK39096); A.laibachii_1_CCA16972: Albugo laibachii Nc14 serine protease family S08A, putative, aa 475–730 (CCA16972); A.laibachii_2_CCA21883: A. laibachii Nc14 serine protease family S08A, putative, aa 162–399 (CCA21883); P.infestans_1_XP_002897506: Phytophthora infestans serine protease family S08A, putative, aa 187–425 (XP002897506); P.infestans_2_XP_002909265: P. infestans serine protease family S08A, putative, aa 141–385 (XP_002909265); P.infestans_3_XP_002901381: P. infestans serine protease family S08A, putative, aa 195–433 (XP002901381); P.infestans_4_XP_002901382: P. infestans serine protease family S08A, putative, aa 196–399 (XP_002901382); P.sojae_1_EGZ19243: Phytophthora sojae subtilisin serine protease, aa 191–428 (EGZ19243); P.sojae_2_EGZ08708: P. sojae subtilisin serine protease, aa 132–382 (EGZ08708); P.sojae_3_EGZ12954: P. sojae subtilisin serine protease, aa 184–434 (EGZ12954); P.sojae_4_EGZ15546: P. sojae hypothetical protein PHYSODRAFT_509390, aa 143–396 (EGZ15546); L.giganteum_ABY90127: Lagenidium giganteum subtilisin-like serine protease, aa 1–174 (ABY90127); M.sp_ZY_04604370: Micromonospora sp. ATCC 39149 peptidase S8 and S53 subtilisin kexin sedolisin, aa 191–444 (ZP04604370); M.lupini_ZP_21031234: Micromonospora lupini str. Lupac 08Peptidase S8 and S53 subtilisin kexin sedolisin, aa 194–454 (ZP21031234); M.aurantiaca_YP_003835151: Micromonospora aurantiaca peptidase S8 and S53 subtilisin kexin sedolisin, aa 191–451 (YP003835151). ‘*’ indicates that the residues or nucleotides in that column are identical in all sequences in the alignment; ‘:’ indicates that conserved substitutions have been ob [file mmc2.pptx]

## Slide 1
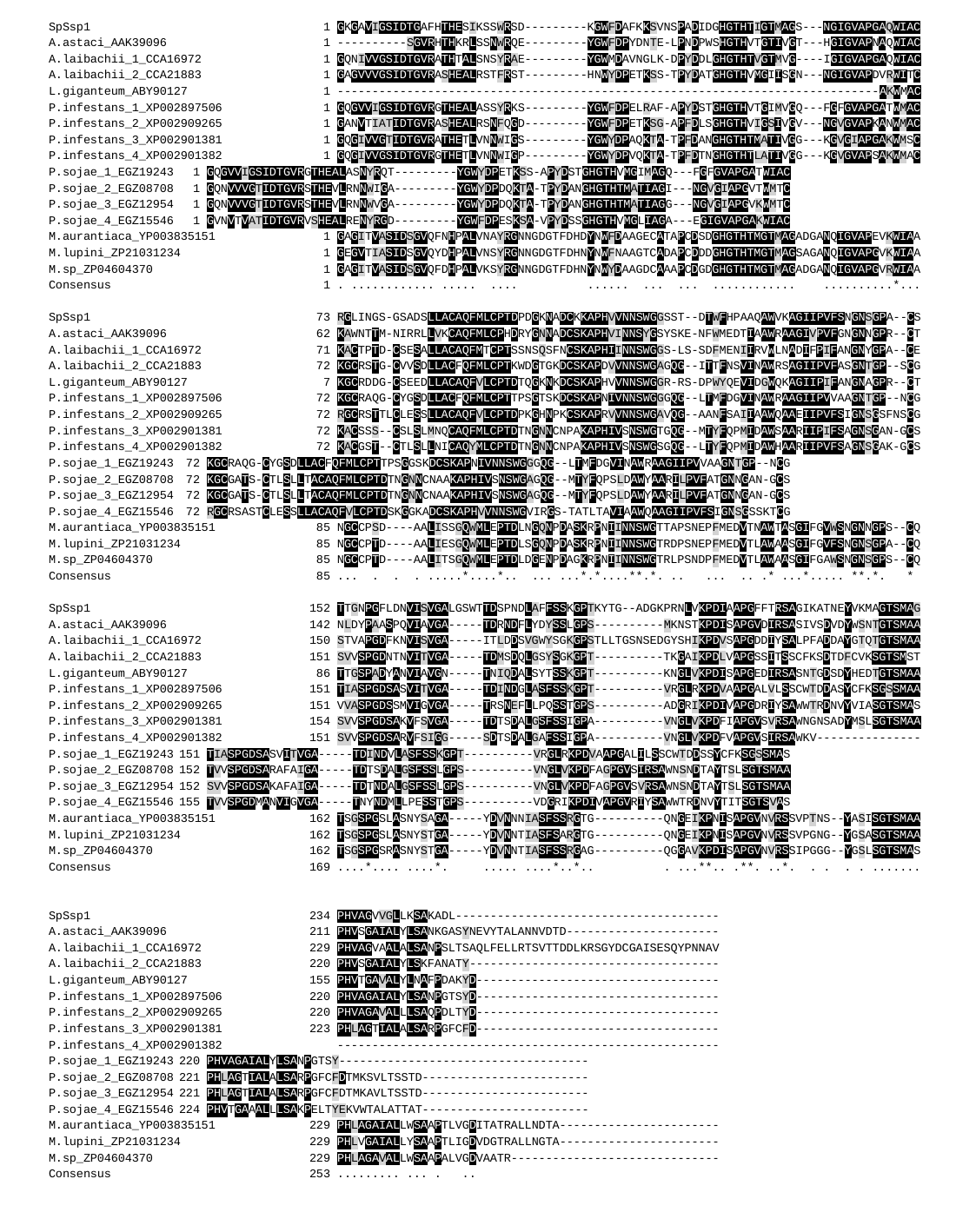

SpSsp1		 1 GKGAVIGSIDTGAFHTHESIKSSWRSD---------KGWFDAFKKSVNSPADIDGHGTHTIGTMAGS---NGIGVAPGAQWIAC
A.astaci_AAK39096		 1 ----------SGVRHTHKRLSSNWRQE---------YGWFDPYDNTE-LPNDPWSHGTHVTGTIVGT---HGIGVAPNAQWIAC
A.laibachii_1_CCA16972	 1 GQNIVVGSIDTGVRATHTALSNSYRAE---------YGWMDAVNGLK-DPYDDLGHGTHTVGTMVG----IGIGVAPGAQWIAC
A.laibachii_2_CCA21883	 1 GAGVVVGSIDTGVRASHEALRSTFRST---------HNWYDPETKSS-TPYDATGHGTHVMGIISGN---NGIGVAPDVRWITC
L.giganteum_ABY90127	 1 ------------------------------------------------------------------------------AKWMAC
P.infestans_1_XP002897506	 1 GQGVVIGSIDTGVRGTHEALASSYRKS---------YGWFDPELRAF-APYDSTGHGTHVTGIMVGQ---FGFGVAPGATWMAC
P.infestans_2_XP002909265	 1 GANVTIATIDTGVRASHEALRSNFQGD---------YGWFDPETKSG-APFDLSGHGTHVIGSIVGV---NGVGVAPKANWMAC
P.infestans_3_XP002901381	 1 GQGIVVGTIDTGVRATHETLVNNWIGS---------YGWYDPAQKTA-TPFDANGHGTHTMATIVGG---KGVGIAPGAKWMSC
P.infestans_4_XP002901382	 1 GQGIVVGSIDTGVRGTHETLVNNWIGP---------YGWYDPVQKTA-TPFDTNGHGTHTLATIVGG---KGVGVAPSAKWMAC
P.sojae_1_EGZ19243	 1 GQGVVIGSIDTGVRGTHEALASNYRQT---------YGWYDPETKSS-APYDSTGHGTHVMGIMAGQ---FGFGVAPGATWIAC
P.sojae_2_EGZ08708	 1 GQNVVVGTIDTGVRSTHEVLRNNWIGA---------YGWYDPDQKTA-TPYDANGHGTHTMATIAGI---NGVGIAPGVTWMTC
P.sojae_3_EGZ12954	 1 GQNVVVGTIDTGVRSTHEVLRNNWVGA---------YGWYDPDQKTA-TPYDANGHGTHTMATIAGG---NGVGIAPGVKWMTC
P.sojae_4_EGZ15546	 1 GVNVTVATIDTGVRVSHEALRENYRGD---------YGWFDPESKSA-VPYDSSGHGTHVMGLIAGA---EGIGVAPGAKWIAC
M.aurantiaca_YP003835151	 1 GAGITVASIDSGVQFNHPALVNAYRGNNGDGTFDHDYNWFDAAGECATAPCDSDGHGTHTMGTMAGADGANQIGVAPEVKWIAA
M.lupini_ZP21031234	 1 GEGVTIASIDSGVQYDHPALVNSYRGNNGDGTFDHNYNWFNAAGTCADAPCDDDGHGTHTMGTMAGSAGANQIGVAPGVKWIAA
M.sp_ZP04604370		 1 GAGITVASIDSGVQFDHPALVKSYRGNNGDGTFDHNYNWYDAAGDCAAAPCDGDGHGTHTMGTMAGADGANQIGVAPGVRWIAA
Consensus		 1 . ............ ..... .... ...... ... ... ............ ..........*...
SpSsp1		 73 RGLINGS-GSADSLLACAQFMLCPTDPDGKNADCKKAPHVVNNSWGGSST--DTWFHPAAQAWVKAGIIPVFSNGNSGPA--CS
A.astaci_AAK39096		 62 KAWNTTM-NIRRLLVKCAQFMLCPHDRYGNNADCSKAPHVINNSYGSYSKE-NFWMEDTIAAWRAAGIVPVFGNGNNGPR--CT
A.laibachii_1_CCA16972	 71 KACTPTD-CSESALLACAQFMTCPTSSNSQSFNCSKAPHIINNSWGGS-LS-SDFMENIIRVWLNADIFPIFANGNYGPA--CE
A.laibachii_2_CCA21883	 72 KGCRSTG-CVVSDLLACFQFMLCPTKWDGTGKDCSKAPDVVNNSWGAGQG--ITTFNSVINAWRSAGIIPVFASGNTGP--SCG
L.giganteum_ABY90127	 7 KGCRDDG-CSEEDLLACAQFVLCPTDTQGKNKDCSKAPHVVNNSWGGR-RS-DPWYQEVIDGWQKAGIIPIFANGNAGPR--CT
P.infestans_1_XP002897506	 72 KGCRAQG-CYGSDLLACFQFMLCPTTPSGTSKDCSKAPNIVNNSWGGGQG--LTMFDGVINAWRAAGIIPVVAAGNTGP--NCG
P.infestans_2_XP002909265	 72 RGCRSTTLCLESSLLACAQFVLCPTDPKGHNPKCSKAPRVVNNSWGAVQG--AANFSAIIAAWQAAEIIPVFSIGNSGSFNSCG
P.infestans_3_XP002901381	 72 KACSSS--CSLSLMNQCAQFMLCPTDTNGNNCNPAKAPHIVSNSWGTGQG--MTYFQPMIDAWSAARIIPIFSAGNSGAN-GCS
P.infestans_4_XP002901382	 72 KACGST--CTLSLLNICAQYMLCPTDTNGNNCNPAKAPHIVSNSWGSGQG--LTYFQPMIDAWHAARIIPVFSAGNSGAK-GCS
P.sojae_1_EGZ19243	 72 KGCRAQG-CYGSDLLACFQFMLCPTTPSGGSKDCSKAPNIVNNSWGGGQG--LTMFDGVINAWRAAGIIPVVAAGNTGP--NCG
P.sojae_2_EGZ08708	 72 KGCGATS-CTLSLLTACAQFMLCPTDTNGNNCNAAKAPHIVSNSWGAGQG--MTYFQPSLDAWYAARILPVFATGNNGAN-GCS
P.sojae_3_EGZ12954	 72 KGCGATS-CTLSLLTACAQFMLCPTDTNGNNCNAAKAPHIVSNSWGAGQG--MTYFQPSLDAWYAARILPVFATGNNGAN-GCS
P.sojae_4_EGZ15546	 72 RGCRSASTCLESSLLACAQFVLCPTDSKGGKADCSKAPHVVNNSWGVIRGS-TATLTAVIAAWQAAGIIPVFSIGNSGSSKTCG
M.aurantiaca_YP003835151	 85 NGCCPSD----AALISSGQWMLEPTDLNGQNPDASKRPNIINNSWGTTAPSNEPFMEDVTNAWTASGIFGVWSNGNNGPS--CQ
M.lupini_ZP21031234	 85 NGCCPTD----AALIESGQWMLEPTDLSGQNPDASKRPNIINNSWGTRDPSNEPFMEDVTLAWAASGIFGVFSNGNSGPA--CQ
M.sp_ZP04604370		 85 NGCCPTD----AALITSGQWMLEPTDLDGENPDAGKRPNIINNSWGTRLPSNDPFMEDVTLAWAASGIFGAWSNGNSGPS--CQ
Consensus		 85 ... . . . .....*....*.. ... ...*.*....**.*. .. ... .. .* ...*..... **.*. *
SpSsp1		152 TTGNPGFLDNVISVGALGSWTTDSPNDLAFFSSKGPTKYTG--ADGKPRNLVKPDIAAPGFFTRSAGIKATNEYVKMAGTSMAG
A.astaci_AAK39096		142 NLDYPAASPQVIAVGA-----TDRNDFLYDYSSLGPS----------MKNSTKPDISAPGVDIRSASIVSDVDYWSNTGTSMAA
A.laibachii_1_CCA16972	150 STVAPGDFKNVISVGA-----ITLDDSVGWYSGKGPSTLLTGSNSEDGYSHIKPDVSAPGDDIYSALPFADDAYGTQTGTSMAA
A.laibachii_2_CCA21883	151 SVVSPGDNTNVITVGA-----TDMSDQLGSYSGKGPT----------TKGAIKPDLVAPGSSITSSCFKSDTDFCVKSGTSMST
L.giganteum_ABY90127	 86 TTGSPADYANVIAVGN-----TNIQDALSYTSSKGPT----------KNGLVKPDISAPGEDIRSASNTGDSDYHEDTGTSMAA
P.infestans_1_XP002897506	151 TIASPGDSASVITVGA-----TDINDGLASFSSKGPT----------VRGLRKPDVAAPGALVLSSCWTDDASYCFKSGSSMAA
P.infestans_2_XP002909265	151 VVASPGDSSMVIGVGA-----TRSNEFLLPQSSTGPS----------ADGRIKPDIVAPGDRIYSAWWTRDNVYVIASGTSMAS
P.infestans_3_XP002901381	154 SVVSPGDSAKVFSVGA-----TDTSDALGSFSSIGPA----------VNGLVKPDFIAPGVSVRSAWNGNSADYMSLSGTSMAA
P.infestans_4_XP002901382	151 SVVSPGDSARVFSIGG-----SDTSDALGAFSSIGPA----------VNGLVKPDFVAPGVSIRSAWKV---------------
P.sojae_1_EGZ19243	151 TIASPGDSASVITVGA-----TDINDVLASFSSKGPT----------VRGLRKPDVAAPGALILSSCWTDDSSYCFKSGSSMAS
P.sojae_2_EGZ08708	152 TVVSPGDSARAFAIGA-----TDTSDALGSFSSLGPS----------VNGLVKPDFAGPGVSIRSAWNSNDTAYTSLSGTSMAA
P.sojae_3_EGZ12954	152 SVVSPGDSAKAFAIGA-----TDTNDALGSFSSLGPS----------VNGLVKPDFAGPGVSVRSAWNSNDTAYTSLSGTSMAA
P.sojae_4_EGZ15546	155 TVVSPGDMANVIGVGA-----TNYNDMLLPESSTGPS----------VDGRIKPDIVAPGVRIYSAWWTRDNVYTITSGTSVAS
M.aurantiaca_YP003835151	162 TSGSPGSLASNYSAGA-----YDVNNNIASFSSRGTG----------QNGEIKPNISAPGVNVRSSVPTNS--YASISGTSMAA
M.lupini_ZP21031234	162 TSGSPGSLASNYSTGA-----YDVNNTIASFSARGTG----------QNGEIKPNISAPGVNVRSSVPGNG--YGSASGTSMAA
M.sp_ZP04604370		162 TSGSPGSRASNYSTGA-----YDVNNTIASFSSRGAG----------QGGAVKPDISAPGVNVRSSIPGGG--YGSLSGTSMAS
Consensus		169 ....*.... ....*. ..... ....*..*.. . ...**.. .**. ..*. . . . . .......
SpSsp1		234 PHVAGVVGLLKSAKADL--------------------------------------
A.astaci_AAK39096		211 PHVSGAIALYLSANKGASYNEVYTALANNVDTD----------------------
A.laibachii_1_CCA16972	229 PHVAGVAALALSANPSLTSAQLFELLRTSVTTDDLKRSGYDCGAISESQYPNNAV
A.laibachii_2_CCA21883	220 PHVSGAIALYLSKFANATY------------------------------------
L.giganteum_ABY90127	155 PHVTGAVALYLNAFPDAKYD-----------------------------------
P.infestans_1_XP002897506	220 PHVAGAIALYLSANPGTSYD-----------------------------------
P.infestans_2_XP002909265	220 PHVAGAVALLLSAQPDLTYD-----------------------------------
P.infestans_3_XP002901381	223 PHLAGTIALALSARPGFCFD-----------------------------------
P.infestans_4_XP002901382	 -------------------------------------------------------
P.sojae_1_EGZ19243	220 PHVAGAIALYLSANPGTSY------------------------------------
P.sojae_2_EGZ08708	221 PHLAGTIALALSARPGFCFDTMKSVLTSSTD------------------------
P.sojae_3_EGZ12954	221 PHLAGTIALALSARPGFCFDTMKAVLTSSTD------------------------
P.sojae_4_EGZ15546	224 PHVTGAAALLLSAKPELTYEKVWTALATTAT------------------------
M.aurantiaca_YP003835151	229 PHLAGAIALLWSAAPTLVGDITATRALLNDTA-----------------------
M.lupini_ZP21031234	229 PHLVGAIALLYSAAPTLIGDVDGTRALLNGTA-----------------------
M.sp_ZP04604370		229 PHLAGAVALLWSAAPALVGDVAATR------------------------------
Consensus		253 ......... ... . ..
